# Case of Tic Douloureux, Cured by the Extraction of a Tooth

**Published:** 1831-03

**Authors:** D. Barry


### TIC DOULOUREUX.

### *Case of Tic Douloureux, cured by the Extraction of a Tooth.* By Dr. D. Barry.

*To the Editor of the London Medical and Physical Journal.*

Mr. N., aged forty-eight, of good, though not very robust constitution, of highly developed mental energies, and the father of a large, healthy family, arrived in this country last spring, from the south of Europe, where he had resided for ten years, with little or no intermission. He had visited most of the great commercial towns in the empire, since his return to England, and whilst at one of the fashionable watering places, about three months ago, consulted a distinguished surgeon for an eruption on his legs, which then, as on a former, though distant occasion, produced a painful itching, with a slight vesicular, serous discharge. He was ordered to dust, or dredge, the little sores with powdered lapis calaminaris, enclosed in a fine muslin bag. This was accordingly done, and within thirty hours the whole of the sores were perfectly dried up: but, after a very few hours more, the inguinal glands on both sides swelled, became hot and painful, and eventually suppurated. When this event took place, Mr. N. was in Bath, where, under mild, aperient, soothing treatment, continued for about six weeks, he recovered. No mercury was used, except as an external application, in the shape of a dilute solution of the muriate in almond milk, to a small superficial ulcer, which had appeared on the inner surface of the prepuce, long after the glands in the groin had suppurated.

About the time (Mr. N., upon being questioned, thinks before the time,) that this lotion was first applied, he was attacked, at two o'clock in the morning, by an excruciating pain in the right half of the lower jaw, extending to the ear, the temple, and right eye, with a ringing noise of a most distressing nature, in that side of the head. This pain returned regularly, every morning, about the same time, and, after continuing for three or four hours, left him; when he usually had some short, but not refreshing sleep. Under these circumstances, Mr. N. came to London; and, though his suffering bore no resemblance to toothach, which he had experienced before, he consulted an eminent dentist, as he felt the gum painful and swelled on the affected side, from which the two anterior molares had, almost spontaneously, come away some years ago, by absorption of the roots and obliteration of the sockets, without pain. The dentist observed a slight oozing of pus between the inner gum and the two remaining molares; but, as these teeth appeared to be perfectly sound, and there being no toothach, he merely scarified the gums. This operation relieved the heat and swelling of the part, but produced no change whatever in the periodical agonies with which Mr. N. had now been regularly visited every morning, for more than a month, and to which he and his friends had applied the dreadful name of *Tic douloureux*.

It was at this period of the disease that I had the honour of being consulted. Having heard the above details, and examined the parts affected, I recommended Ol. Amygdal. dul. with Tinct. Opii, to the meatus externus, where I thought that I could perceive a deficiency of cerumen; warm fomentations to the cheek, cupping to ten ounces at the back of the neck, and an aperient. These measures, which, as I informed the patient, were only preparatory to the administration of quinine, were all faithfully adopted; but, as I could perceive next morning, they only left the sufferer more prostrate, without having alleviated his agonies in the slightest degree.

The sulphate of quinine was now prescribed, in doses of two grains three times in twelve hours. On the morning of the day after this medicine had been taken, the pain came on rather earlier, and was, if possible, more severe than usual. When I saw him about one o'clock P.M., he complained that he felt more uncomfortable than he had done, during the day, since the commencement of this disease.

Two collateral circumstances ought, perhaps, to be mentioned here.

1st. That Mr. N., at my first visit, cautioned me not to mix opium, nor indeed any narcotic, with his medicine, as that class of drugs affected him in a peculiarly distressing way. The drop or two of laudanum put into his ear, with his own consent, had, he said, rendered him so miserable, that he should never again admit a repetition of that remedy.

2d. When I first saw Mr. N., the little ulcer on the prepuce was still open, and the mercurial lotion was still continued. Though three days only had elapsed since this lotion had been laid aside at my request, and frequent fomentations of Infus. Anthemed. nob., with dry lint dressing, substituted, the ulcer was found to be perfectly healed.

At the fourth visit, the teeth were again examined, when the anterior of the two molares, already noticed, was fully ascertained to be a little loose in its socket, although alternate pressure, made on either side of it, gave no pain. After reasoning on the possibility of this tooth being the cause of all the suffering, it was settled, to give the quinine another day's trial, but, if it again failed to afford relief, that the tooth should be extracted.

Upon calling next day, I found my patient looking heavy and disappointed. The attack of that morning had been dreadful: the pain had, most unusually, he said, threatened him so early as eleven o'clock at night; when, in a fit of despair, he ordered a mutton-chop, highly peppered, which he ate, took some brandy and water hot and strong, and by those means kept the enemy at bay, as he asserted, until five o'clock in the morning. This paroxysm, however, was of more than ordinary violence.

The tooth was now ordered to be extracted immediately, and a warm bath to be taken after the operation: all other treatment to be discontinued.

The dentist, upon re-examining the condemned tooth, declared that it presented no visible motive for extraction, that the inflammation of the gum had disappeared, and that he did not consider himself justified in removing an apparently sound tooth. Mr. N., however, was firm, and had it taken out. The operator, upon presenting the tooth to the patient, observed, that he no longer regretted the operation, as he could see that one of the fangs was carious. This was on the 5th instant, and Mr. N. has continued ever since free from pain of any kind.*

I send you the tooth itself, in which you will perceive some fine, sharp spiculæ of carious bone, just at the point of one of the fangs. The other root is sound, and was perfectly adherent to the lining of its alveola.†

I need not point out to you how strongly this case corroborates the practical views brought before the profession, in a valuable paper on Tic douloureux, by Sir H. HALFORD, read in 1828, at one of the conversaziones of the College of Physicians. In that paper, the learned President adduced many striking instances from his own extensive experience, and other high authorities, proving that the cause of Tic, particularly of its most severe forms, which usually affect the branches of the fifth pair of nerves, is often demonstrable to the senses even before death, and consists in osseous disease.

The means of *curing* or *alleviating disease*, the great end and aim of all our professional studies, though in a great measure due to the accumulated results of prescriptionary experiment, have been of late years largely increased by the researches of morbid anatomy. The long catalogue of mala-

* Dr. Barry met Mr. N. yesterday (14th February), in perfect health; nor had he experienced any return of the Tic since the tooth had been extracted.—D.B.

† Dr. Barry has shown us the tooth: the description he gives of its appearance is perfectly correct.—EDITOR.

dies, hitherto vaguely termed idiopathic, has been much abridged. We have learnt that constitutional disturbance may be produced by individual organic derangement, and that, by removing the local cause of irritation, we can give calm and health to the general system.

*Welbeck street; 15th January* 1831.

